# Fasciclin-calcareous corpuscle binary complex mediated protein-protein interactions in *Taenia solium* metacestode

**DOI:** 10.1186/s13071-017-2359-2

**Published:** 2017-09-20

**Authors:** Chun-Seob Ahn, Jeong-Geun Kim, Young-An Bae, Seon-Hee Kim, Joo-Ho Shin, Yichao Yang, Insug Kang, Yoon Kong

**Affiliations:** 1 Department of Molecular Parasitology, Samsung Medical Center, Sungkyunkwan University School of Medicine, Suwon, 16419 South Korea; 2 Department of Microbiology, Gachon University College of Medicine, Incheon, 21936 South Korea; 3 Division of Pharmacology, Department of Molecular Cell Biology, Samsung Medical Center, Sungkyunkwan University School of Medicine, Suwon, 16419 South Korea; 4 Guangxi Centers for Disease Prevention and Control, Nanning, Guangxi 53002 China; 5 Department of Molecular Biology and Biochemistry, School of Medicine, Kyung Hee University, Seoul, 02447 South Korea

**Keywords:** *Taenia solium* metacestode, Neurocysticercosis, Calcareous corpuscle, Fasciclin, Carbohydrate metabolizing enzyme, Extracellular matrix, Protein-protein interactions

## Abstract

**Background:**

Neurocysticercosis (NC) caused by *Taenia solium* metacestode (TsM) is a serious neurological disease of global concern. Diverse bioactive molecules involved in the long-term survival of TsM might contribute to disease progression. Fasciclin (Fas) is an extracellular protein that mediates adhesion, migration and differentiation of cells by interacting with other molecules. We hypothesized that TsMFas might bind to calcareous corpuscle (CC) through its adhesive property and participate in crucial protein-protein interactions, thus contributing to the creation of a symbiotic interactome network.

**Methods:**

Two paralogous TsMFas (TsMFas1 and TsMFas2) were isolated, and their molecular properties were characterized. The co-localization pattern of TsMFas1 and TsMFas2 with CC was determined. CC-TsMFas binary complex was generated by incubating CC with recombinant proteins (rTsMFas1 and 2). In vitro binding assay of CC-rTsMFas1 or CC-rTsMFas2 binary complex with TsM cellular proteins extracted from scolex and neck was conducted. Their binding partners were identified through proteomic analysis. Integrated protein-protein interaction networks were established.

**Results:**

*TsMFas1* (6072 bp long) was composed of 15 exons (841 amino acid polypeptide) interrupted by 14 introns. *TsMFas2* (5201 bp long) comprised of 11 exons (597 amino acids) and 10 intervening introns. These proteins displayed 22% amino acid sequence identity to each other, but tightly conserved Fas-related domains. Several isoforms of Fas1 and Fas2 proteins might have been expressed through post-translational modifications. They showed adhesion activity with other cells. TsMFas proteins were largely distributed in parenchymal regions of the scolex and bladder wall. These molecules were co-localized with CC, a unique organelle found in platyhelminths. Subsequent proteome analysis of CC-Fas binary complex mediated protein-protein interactions revealed seven protein ligands in the TsM cellular proteins. Their functions were mainly segregated into carbohydrate metabolism (enolase, phosphoenolpyruvate carboxykinase, phosphoglycerate kinase and glyceraldehyde 3-phosphate dehydrogenase) and cytoskeleton/cellular motility (actin, paramyosin and innexin nuc-9). Those proteins had direct (physical) and/or indirect (functional) relationships along with their biochemical properties and biological roles.

**Conclusion:**

Protein repertoires strongly suggest that TsMFas and CC may symbiotically mediate protein-protein interactions during biological processes to maintain efficacious homeostatic functions and ensure the prolonged survival of TsM in the host.

**Electronic supplementary material:**

The online version of this article (10.1186/s13071-017-2359-2) contains supplementary material, which is available to authorized users.

## Background

Neurocysticercosis (NC) is an infection of the central nervous system (CNS) with the *Taenia solium* metacestode (TsM). The disease is detected worldwide, but is prevalent in several countries of Southeast Asia, China, Central/Latin America, the Indian subcontinent and sub-Saharan Africa [1, 2]. Seizure disorders related to NC have been estimated to have 1.7 to 3 million annually in areas where the disease is endemic. Approximately 50 million people are at risk, and 50,000 die each year due to NC. Epidemiological evidence indicated that increasing tendency of childhood NC worsens disability-adjusted life years (DALY) [3, 4]. NC is an important neglected tropical disease due to its significant disease burdens and impacts on DALY associated with social stigmatization and economic losses (http://www.who.int/neglected_diseases/diseases/en/).

Clinical manifestations of NC are highly heterogeneous according to the location, numbers and viability of the worms in the brain, but principal symptoms include headache, seizure and other focal neurologic deficits. Acute and/or chronic inflammations and hydrocephalus induced by the invasive parasite are the main pathogenetic factors associated with clinical presentations [4–6]. TsM can thrive in immunocompetent human hosts for more than five years and causes significant pathobiological alterations. This result suggests that NC might not result from a simple infection of TsM, but a complex phenomenon mediated by cell biological cross-talk between TsM and the host [7]. TsM has to continuously synthesize bioactive molecules to adapt to the hostile host environment to ensure its prolonged survival through maintaining cellular homeostatic functions [7, 8].

Proteins harbour a single and/or multiple fasciclin (Fas) 1 and Fas-superfamily domains are found in a wide range of eukaryotes. Those molecules are mostly membrane-anchored glycoproteins and are expressed on the cell surface. They participate in homophilic cell adhesion and symbiotic processes [9–12]. Fas1/transforming growth factor-β-induced gene (TGFBI/βig-h3) containing proteins interact with integrin and mediate migration of extracellular matrix (ECM) molecules in different types of cells [10, 13]. Helminth fasciclins have been identified and characterized in *Schistosoma mansoni*. The protein, initially known as a gynecophoral canal protein (GCP), is abundantly expressed in gynecophoral canal regions of the male worm. The protein is not only involved in cellular adhesion, migration and differentiation of the worm [14] but also in the development of the male-induced female reproductive system by stimulating host TGF-β signalling [15]. *Paragonimus westermani* Fas1-domain containing protein (Pwfas-I) adhered to ανβ5 integrin within ECM molecules. The protein is localized to the contact region of paired worms, suggesting its roles during worm’s development and subsequent mating [11].

One of the unique histological features of platyhelminth parasites is the presence of numerous mineralized concretions, termed calcareous corpuscles (CCs), in cellular parenchyma [16]. CCs are mostly synthesized under anaerobic conditions [17]. They are composed of biomineral components (calcium, magnesium, manganese and copper) and organic matrices comprised of DNA/RNA, lipids, carbohydrates, proteins and glycosaminoglycans [18]. The spheroid or ovoid organelle consists of two layers of internal concentric and outer corpuscle-forming layers. CC is thought to be involved in the focal accumulation of ions and heavy metals, osmoregulation and protection of parasites against calcification [19]. CC has binding affinity to glycoproteins and calcium-binding proteins [20–23]. A recent study with *Echinococcus granulosus* metacestode has demonstrated that CC might undergo active catabolic process during drug-induced autophagy [24]. These results collectively suggest that CC might have functional roles in cellular events during long-term survival of the parasite. However, the biological relevance of CC remains unclear.

In the present study, we hypothesized that TsMFas might bind to CC and mediate crucial protein-protein interactions, thus contribute to the creation of a symbiotic interactome network. To explore such protein interactions, we analyzed protein repertoires bound to CC. Several proteins involved in cellular/organismal processes and cellular components and molecules associated with biochemical pathways were shown to bind to CC. TsMFas1 and TsMFas2 proteins also bound to CC. We subsequently analyzed CC-TsMFas binary complex mediated protein-protein interactions. Our results demonstrated that enzymes involved in carbohydrate metabolism and proteins composing cytoskeleton/cellular motility were specifically interactive.

## Methods

### Parasites

TsM and adult worms were collected from naturally infected pigs and humans in an endemic area (Tiandong County, Guangxi Zhuang Autonomous Province, China). Adult tapeworms were identified by observing a diagnostic 474 bp long fragment that appeared in PCR [25]. Intact TsM cysts were individually collected from pig muscles, after which cyst fluid (CF) was drained using an aseptic 26 gage-needle in the presence of protease inhibitor cocktail (Complete; Roche, Penzberg, Germany). One tablet was applied per 25 ml CF. Excretory-secretory products (ESP) were obtained by incubating adult (single worm) and metacestode (50 intact TsM) in serum-free RPMI-1640 media (pH 7.2; Life Technology, Waltham, MA, USA) for 2 h at 37 °C [26]. Samples were separately prepared according to individual anatomical compartments such as scolex/neck (adult and TsM), immature, mature and gravid proglottids (adult) and bladder wall (TsM). Each sample was homogenized using a Teflon-pestle homogenizer in 100 mM phosphate buffered saline (PBS, pH 7.4) containing protease inhibitor cocktail. All samples including CF and ESP were centrifuged at 20,000× *g* for 1 h at 4 °C. Resultant supernatants were stored at -80 °C.

### Cloning and sequence analysis

Two paralogous genes putatively coding for TsMFas (*TsMFas1* and *TsMFas2*) were retrieved from GeneDB (TsM_000655200 and TsM_000825900) (http://www.genedb.org/Homepage/Tsolium). Nucleotide sequences corresponding to mature domains of TsMFas1 and TsMFas2 (amino acid residues between 23 and 841 and between 38 and 597, respectively) were verified with Signal-BLAST (http://sigpep.services.came.sbg.ac.at/signalblast.html) and SignalP 4.1 Server (http://www.cbs.dtu.dk/services/SignalP/). These genes were amplified with a TsM cDNA library [5] using specific primers (*Bam*HI-tagged forward primer 5′-CCG GGG ATC CGC TCT CAA CCT TAC CAC C-3′ and *Not*I-tagged reverse primer 5′-GCG GCC GCC TAG TGT TCG TGA CCC TC-3′ for *TsMFas1*; *Hin*dIII-tagged forward primer 5′-CGC AAG CTT ATG GCG CCC GAG CTA-3′ and *Xho*I-tagged reverse primer 5′-CGC CTC GAG TTT AGC AGT TGT TAC AGA C-3′ for *TsMFas2*) (restriction sequences for each enzyme are underlined). PCR amplifications were done with cycling parameters of 95 °C for 5 min, 35 cycles at 95 °C for 1 min, 55 °C for 1 min, 72 °C for 3 min and a final extension at 72 °C for 10 min. Amplicons were digested with restriction enzymes, ligated into pET-28a(+) vector (Novagen, Cambridge, MA, USA) and transformed into *Escherichia coli* BL21 (DE3). Their sequences were confirmed by sequencing using ABI BigDye Terminator ver3.1 with cycle sequencing kits.

The domain structures were examined with NCBI Conserved Domain Search (http://www.ncbi.nlm.nih.gov/Structure/cdd/wrpsb.cgi) and InterProScan 4 (http://www.ebi.ac.uk/Tools/pfa/iprscan/). Phosphorylation sites were predicted by NetPhos 3.1 (http://www.cbs.dtu.dk/services/NetPhos/). N- and O-glycosylation sites were analyzed by NetNGlyc 1.0 (http://www.cbs.dtu.dk/services/NetNGlyc/) and NetOGlyc 4.0 (http://www.cbs.dtu.dk/services/NetOGlyc/), respectively.

### Phylogenetic analysis

Fasiclin-like proteins were retrieved during BLASTp and tBLASTn searches of non-redundant genomic and proteomic databases of GenBank with query sequence of TsMFas1 and TsMFas2. Chordata (Taxid: 7711), Arthropoda (Taxid: 6656), Bacteroidetes (Taxid: 976), Apicomplexa (Taxid: 5794), Cestoda (Taxid: 6199) and Trematoda (Taxid: 6178) were included. Arbitrarily, proteins were selected from each taxon by considering identity and expected values. Two orthologous proteins that found in *Taenia saginata* by blast search in WormBase ParaSite (http://parasite.wormbase.org/Multi/Tools/Blast?db=core) were also included [27]. Amino acid sequences of selected proteins were aligned with ClustalW and applied in a phylogenetic analysis using the maximum-likelihood algorithm of Mega 6.0 (http://www.megasoftware.net/) (Jones-Thornton-Tayler model for amino acid substitution, rate heterogeneity with 4 gamma category). Gaps were introduced in the alignment to increase identity values. Gaps resulting from missing data were deleted in a pairwise manner (cut-off: 95%). Statistical reliability of branching nodes was assessed by the standard bootstrapping test of 1000 replicates.

### Expression and purification of recombinant proteins

*Escherichia coli* BL21 (DE3) cells transformed with *TsMFas1* and *2* plasmid constructs were cultured in Luria-Bertani medium containing kanamycin (50 μg/ml). Recombinant proteins (rTsMFas1 and rTsMFas2) were induced with 1 mM isopropyl-β-D-thiogalactopyranoside (IPTG) for 4 h at 37 °C. Cells were harvested and sonicated. rTsMFas1 and 2 proteins were purified using nickel-nitrilotriacetic acid agarose column chromatography (Ni-NTA; Qiagen, Hilden, Germany). His-tag was removed using Thrombin CleanCleave kit (Sigma-Aldrich, St. Louis, MO, USA). Homogeneity of the purified proteins was monitored by 8% reducing SDS-PAGE followed by Coomassie brilliant blue G-250 (CBB) staining.

### Specific antibodies

Specific antibodies against rTsMFas proteins were generated in 6-week-old female specific pathogen-free BALB/*c* mice. Each protein (30 μg per mouse) was immunized subcutaneously 3 times with Freund’s adjuvants (Sigma-Aldrich) at 2-week intervals. Two weeks after the third immunization, proteins (10 μg each) were additionally boosted through intravenous injection. One week later, blood was collected by heart puncture, and immune sera were obtained by centrifugation at 3000× *g* for 5 min. IgG fractions were isolated by protein G-affinity chromatography and stored at -80 °C.

### SDS-PAGE and 2-dimensional gel electrophoresis (2-DE)

Protein samples were separated by 8% or 15% SDS-PAGE under reducing conditions. Cellular proteins extracted from the scolex/neck (SN, 80 μg) were isoelectrically focused with rehydration buffer (7 M urea, 2 M thiourea, 2% CHAPS, 100 mM dithiothreitol [DTT] and 0.5% ampholyte) on immobilized pH gradient strips (IPG, pH 4–7, 7 cm long; GE Healthcare, Piscataway, NJ, USA) for 25 kVh. The IPG strips were further separated by 8% SDS-PAGE on second-dimension gels. Gels were visualized by CBB staining or further processed with immunoblotting with specific antibodies.

### Immunoblotting

Proteins separated by SDS-PAGE or 2-DE were electrotransferred to nitrocellulose membranes (Santa Cruz Biotechnology, Santa Cruz, CA, USA) for 1–6 h at 4 °C. Membranes were blocked with Tris-buffered saline containing 3% skim milk and 0.05% Tween 20 and probed with anti-rTsMFas1 or anti-rTsMFas2 antibody (each at 1:2000 dilution) overnight at 4 °C. Membranes were further incubated with 1:4000 diluted horse-radish peroxidase (HRP)-conjugated anti-mouse IgG antibody (Cappel, Huntington, CA, USA) for 2 h, after which immunoreactive signals were detected with West-Q Pico enhanced chemiluminescence (ECL) kit (GenDEPOT, Dallas, TX, USA). All images were obtained after 2 min of exposure for quantitative analysis. Images were digitalized with an Epson Perfection V700 Photo Scanner (EPSON, Long Beach, CA, USA).

### Immunohistochemical staining

TsM sections (4 μm thick) were permeabilized in PBS containing 0.5% Triton X-100 for 15 min and incubated in Tris-HCl (10 mM, pH 8.0) supplemented with proteinase K (20 ng/ml) at 37 °C for 15 min. Slides were blocked with PBS containing 0.05% Tween 20 (PBS/T) and 3% bovine serum albumin (BSA) for 1 h followed by incubation with respective antibodies (1:200 dilution) at 4 °C overnight. The slides were subsequently incubated with fluorescein isothiocyanate (FITC)-conjugated goat anti-mouse IgG antibody (1:500 dilution; Abcam, Cambridge, MA, USA) at 4 °C for 1 h. Sections were counterstained with 4′,6-diamidino-2-phenylindole (DAPI, 10 mg/ml; ThermoFisher Scientific, Waltham, MA, USA) at 4 °C for 5 min in the dark. Fluorescent images were visualized using an IX71 inverted microscope (Olympus, Tokyo, Japan). Preimmune mouse serum (IgG fractions) diluted at the same ratio was used as a control.

### Adhesion assay

Micro-culture plates (96-well; NUNC, Sigma-Aldrich) were pre-coated with BSA (2 μg/ml), fibronectin, rTsMFas1 and rTsMFas2 protein (each 10 μg/ml) overnight at 4 °C. Wells were blocked with PBS containing 0.2% BSA at 37 °C for 1 h and washed 3 times with PBS. Normal human lung fibroblast (NHLF; Lonza, Basel, Switzerland) and human fetal lung fibroblast (MRC-5; Sigma-Aldrich) cells were cultured in DMEM containing 10% fetal bovine serum at 37 °C in a 5% CO_2_ incubator. Trypsinized cells (3 × 10^5^/ml) were inoculated into each well (0.1 ml of cell suspension per well) and incubated at 37 °C under 5% CO_2_ atmosphere for 30 min. Unattached cells were removed by washing well with PBS twice. Attached cells were incubated with reaction buffer (50 mM citrate buffer containing 3.75 mM ρ-nitrophenyl-N-acetyl β-D-glucosaminide and 0.25% Triton X-100, pH 5.0) at 37 °C for 1 h. Enzyme activity was blocked by adding 50 mM glycine buffer (pH 10.4) containing 5 mM EDTA. The number of cells was estimated by measuring absorbance value at 405 nm using a microtiter reader (SpectraMax Plus 384; Molecular Devices, Sunnyvale, CA, USA).

### In vitro binding assay

Prior to binding assay, CCs were purified as previously described [22] with some modifications. Briefly, TsM cellular compartments were homogenized in PBS supplemented with protease inhibitor cocktail (Roche) as mentioned above with a Teflon-coated tissue homogenizer. The homogenate was filtered through a cell-strainer (100 μm-pore size). The sample was overlaid on the same volume of Ficoll-Plaque plus (Amersham Biosciences, Uppsala, Sweden) and allowed to stand for 15 min to pellet CCs by gravity. Separated CCs were sequentially washed with PBS/T (5 times) and PBS (5 times). Purified CCs were collected by centrifugation at 1000× *g* for 1 min and resuspended in 100–200 μl of PBS at 4 °C. The purity of isolated CCs was established to be > 95% by microscopic examination. In each experiment, freshly prepared CCs were used.

CC-TsMFas1 or CC-TsMFas2 binary complex was generated by incubating the purified CC (10 μl) with rTsMFas1 or rTsMFas2 protein (each 10 μg) for 2 h on a shaker, after which precipitated by centrifugation at 10,000× *g* for 5 min. The complex was sequentially washed with PBS/T (5 times) and PBS (5 times) followed by centrifugation at 1000× *g* for 1 min. Cellular proteins depleted of Fas1 and 2 molecules were prepared by filtering crude SN extracts through protein G-immunoaffinity chromatography coupled with anti-rTsMFas1 and anti-rTsMFas2 antibodies. Unbound proteins eluted with flow-through fractions were used in binding assays. The CC-TsMFas1 or CC-TsMFas2 binary complex (each 10 μl) was incubated with Fas1 and Fas2 depleted cytosolic proteins (10 μg) overnight. Unbound proteins were washed as above. Samples were centrifuged at 10,000× *g* for 5 min. All procedures were done at 4 °C unless otherwise specified. Bound proteins were resuspended in the same volume of 2× SDS-PAGE reducing sample buffer, boiled for 5 min and separated on 15% gels. Protein bands stained with CBB were sliced from the gel and processed for proteome analysis.

### Mass spectrometry and bioinformatics

Disulfide bonds of proteins were reduced by 10 mM DTT in 25 mM ammonium bicarbonate at 56 °C for 1 h and alkylated with 55 mM iodoacetamide in 25 mM ammonium bicarbonate for 1 h at 25 °C in the dark. Gels were dehydrated with 100% acetonitrile, dried and digested with trypsin in 25 mM ammonium bicarbonate at 37 °C for 6 h. Extracted peptides were dried in a vacuum evaporator (MIVAC DUO; Genevac, Ipswich, UK). Samples were then analyzed by a nano-liquid chromatography-electrospray ionization-multistage mass spectrometry (nano-LC-ESI-MS/MS) using a model 1200 nanoflow system (Agilent Technologies, Palo Alto, CA, USA) connected to an LTQ linear ion trap mass spectrometer (Thermo Electron, San Jose, CA, USA). MS scanning was performed at 300–2000 *m/z* followed by three data-dependent MS/MS scans of isolation width at 1.5 *m/z*, normalized collision energy of 25% and dynamic exclusion duration of 180 s. MS data were generated in a RAW file format using a Xcalibur 1.4 apparatus with Tune 1.0 (Thermo Scientific). Peptide peaks were introduced into MS/MS ions search within Mascot server (http://www.matrixscience.com). Mass values were selected with monoisotopic masses. Peptide and MS/MS tolerances were ±1.2 and ±0.6 Da, respectively. Protein identifications resulting from individual ions scores >37 were considered as significant identity or extensive homology (*P* < 0.05) to the predicted identification displayed. Two modifications (cysteine carbamidomethylation and methionine oxidation) were considered during proteome analyses. MS database search was performed with merged files from *T. solium* GeneDB (http://www.genedb.org/Homepage/Tsolium). Duplicated samples were independently analyzed.

Identified proteins were functionally categorized using Blast2GO ver4 (http://www.blast2go.com) with Blast Expectation Value (E-value = 1.0e-3). InterProScan, mapping, annotation and graphical analyses were sequentially performed using data obtained by BLASTp. Graphical histograms of biological process, molecular function and cellular component were generated with the second-level of GO hierarchy [28]. Physical (direct) and functional (indirect) associations during integrated protein-protein interactions were in silico mapped using STRING ver10.0 algorithm (http://string-db.org/) [29].

### Statistical analysis

Data are expressed as mean ± standard deviations (SD) of three independent experiments. Statistical analyses were done by Student’s *t-*test and one-way analysis of variance (ANOVA) using Statistical Package for Social Sciences software ver20.0 (SPSS, Chicago, IL, USA). Differences in mean values were considered statistically significant at *P* < 0.05.

## Results

### Structural properties of *TsMFas1* and *TsMFas2* genes and phylogenetic analysis

**Fig. 1:**
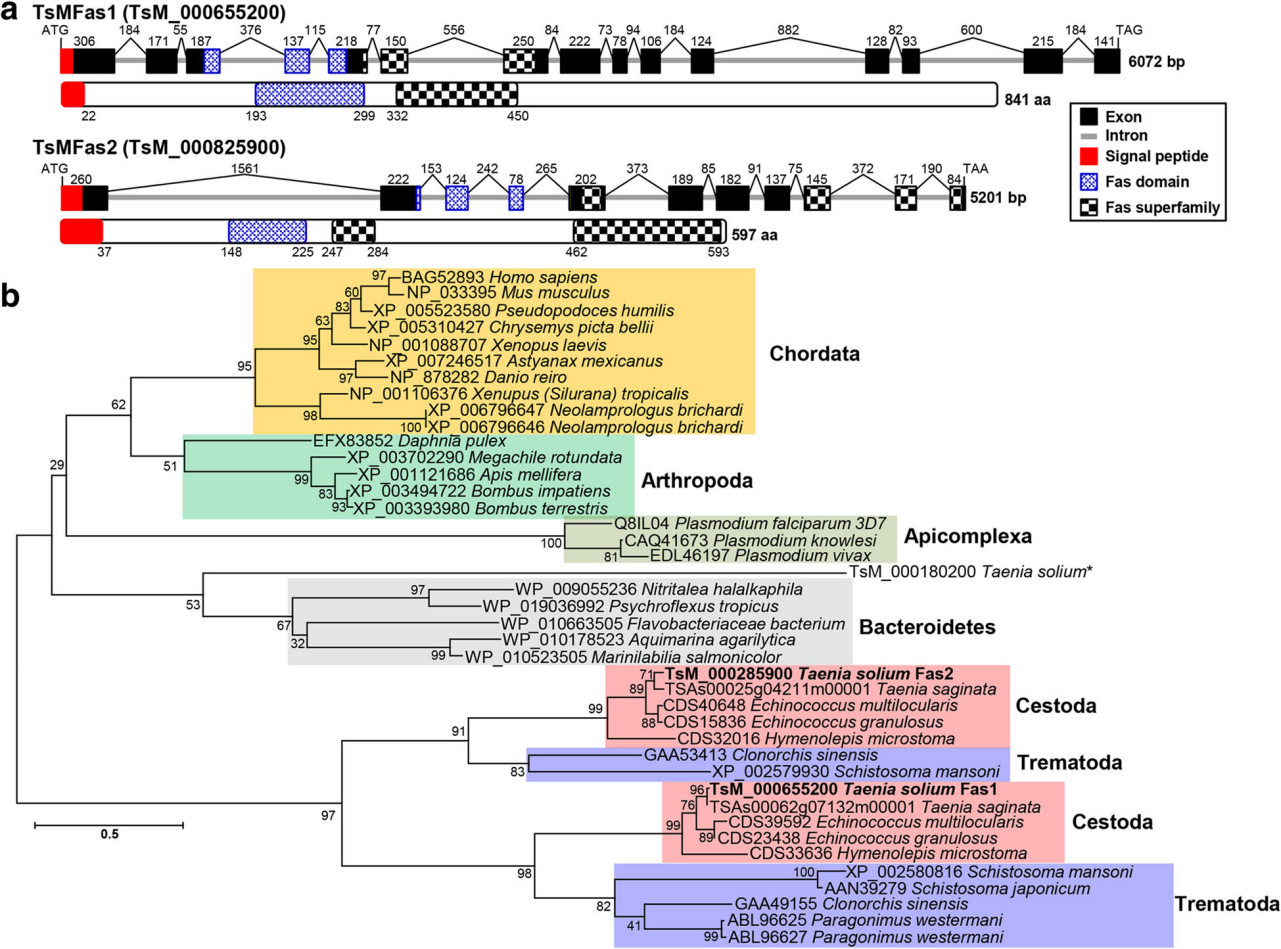
Molecular characteristics of *Taenia solium* metacestode fasciclin (*TsMFas*) genes*.* **a** Schematic representation of exon-intron boundary structure of *TsMFas1* (TsM_000655200) and *TsMFas2* (TsM_000825900) (upper panel) and domain organization of their protein products (lower panel). Exons and introns are marked by black-boxes and gray-solid lines. Numerals on the top indicate exon and intron lengths in bp. Blue and black checkered-boxes matched with fasciclin 1 and fasciclin-superfamily domains, respectively. Red boxes demonstrate signal peptides. **b** The phylogenetic relationships of TsMFas proteins with other 39 fasciclin-domain containing proteins. Maximum-likelihood algorithm (MEGA 6.0) was used for tree construction using full-length amino acid sequences of selected proteins. Numerals at major branching nodes indicate the percentage of appearance in 1000 bootstrap replicates. TsMFas1 and TsMFas2 proteins are highlighted in bold-face. Another fasciclin 1 found in *T. solium* GeneDB (TsM_000180200) is indicated by an asterisk

Coding sequences of *TsMFas1* and *TsMFas2* genes (KC758144 and KC758145) were previously isolated during the investigation of antibody response of TsMFas1 protein against sera from patients with chronic NC [30]. These sequences completely matched with those detected in *T. solium* GeneDB: *TsMFas1* (TsM_000655200) and *TsMFas2* (TsM_000825900). We analyzed molecular characteristics of the genes and proteins. The 6072 bp long *TsMFas1* gene encompassed 2526 bp long coding sequence (841 amino acid polypeptide). It had 15 exons with 14 intervening introns (3546 bp). *TsMFas2* gene (5201 bp long) was composed of 11 exons (1794 bp long for 597 amino acids) interrupted by 10 introns (3407 bp long) (Fig. 1a). These two paralogous proteins showed considerable length polymorphism. Deduced molecular weights (*M*_r_) of TsMFas1 and 2 were 86,956 Da and 65,647 Da with isoelectric point (p*I*) of 5.79 and 5.76, respectively. The two proteins shared relatively low sequence identities of 55% at mRNA level and 22% at the protein level, respectively. However, they both possessed tightly conserved Fas-related domains. TsMFas1 harboured each of single Fas1 and Fas-superfamily domain together with highly-conserved H1 and H2 domains found in several eukaryotic fasciclin molecules [10]. TsMFas2 contained a single Fas1 domain and two Fas-superfamily domains, as well as H1 and H2 domains. The proteins lacked a consensus transmembrane domain or a glycophosphatidylinositol (GPI)-anchoring signal (Additional file 1: Figure S1). Both proteins harboured signal peptide, strongly suggesting that these proteins were secreted through the classical pathway. *M*_r_, of secreted forms was 85,015 Da with p*I* of 5.73 for TsMFas1 and 61,762 Da with p*I* of 5.66 for TsMFas2. Both proteins harboured a couple of phosphorylation sites (Additional file 1: Figure S1, red letters). TsMFas1 contained two N-glycosylation sites (blue letters) while TsMFas2 had none. TsMFas1 and TsMFas2 possessed seven and two putative O-glycosylation sites, respectively (open circles).

We analyzed phylogenetic relationships of TsMFas proteins with their homologs isolated in bacteria, protozoans, platyhelminths, arthropods and vertebrates. Fas-domain containing proteins were tightly co-clustered with one another according to the taxonomic distribution of donor organisms (Fig. 1b). Platyhelminth proteins formed two distinct paralogous Fas1 and Fas2 clades, each of which was comprised of trematode- and cestode-specific subclades, demonstrating a duplication of a common ancestor gene in the platyhelminth lineage at least prior to the divergence of trematodes and cestodes.

During annotation of TsMFas related proteins, one sequence designated as fasciclin 1 could be retrieved from *T. solium* GeneDB (TsM_000180200). However, this protein showed sequence identity as low as 17.1% and 16.3% with TsMFas1 and TsMFas2 proteins. The protein did not have a signal peptide. Sequence producing high-scoring segment pairs also revealed a high probability value of 4.3e-21 between TsMFas1 and TsMFas2, while TsM_000180200 protein showed no probability (data not shown). Phylogenetic analysis also demonstrated that the protein was differently clustered from the cestode clade. The protein showed phylogenetic position close to Bacteroidetes and suggested its earlier divergence than TsMFas1 or TsMFas2 (marked by an asterisk, Fig. 1b). The protein might have a differential evolutionary route to have distinct physical and functional properties.

### Expression of recombinant proteins and adhesion assay

Mature domains of TsMFas1 and TsMFas2 proteins were bacterially expressed and the recombinant proteins were purified using Ni-NTA column followed by thrombin cleavage. The proteins migrated to approximately 85 and 63 kDa, matching well with those predicted by in silico analysis (Additional file 2: Figure S2a). rTsMFas1 and 2 proteins significantly increased adhesions of NHLF (ANOVA: *F*_(4,10)_ = 1802.2, *P* < 0.0001) and MRC-5 (ANOVA: *F*_(4,10)_ = 2309.2, *P* < 0.0001) cells (Additional file 2: Figure S2b). The number of cells adhered to rTsMFas1 or 2 was typically > 80% while that of control cells showed 16–54% of adhesion. This result suggested strongly that both Fas proteins might mediate molecular interactions within cellular parenchyma and that the proteins secreted into surrounding environments might be involved in worm’s attachment to the host tissues.

### Expression profile of Fas proteins in *T. solium*

**Fig. 2:**
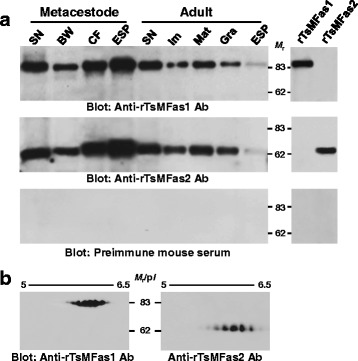
Spatiotemporal expression profile of *T. solium* metacestode fasciclin (TsMFas1 and TsMFas2) proteins. **a** Compartmental expression patterns of TsMFas1 and 2 in *T. solium* metacestode and adult worms. Proteins extracted from individual anatomical compartments and excretory-secretory products (each 10 μg) and recombinant proteins (each 150 ng) were separated by 8% reducing SDS-PAGE, transferred to nitrocellulose membrane and probed with anti-rTsMFas1 or anti-rTsMFas2 antibody, together with preimmune mouse serum (1:2000 dilution). The signal was detected by ECL after 2 min exposure. Lane SN: scolex/neck; Lane BW: bladder wall; Lane CF: cyst fluid; Lane ESP: excretory-secretory products; Lane Im: immature proglottid; Lane Mat: mature proglottid; Lane Gra, gravid proglottid. **b** Expression of different isoforms of TsMFas1 and TsMFas2. SN proteins (80 μg) were isoelectrically focused on IPG strips (pH 4–7, 7 cm long), electrophoresed by 8% SDS-PAGE and electroblotted onto a nitrocellulose membrane. Blots were probed with anti-rTsMFas1 or anti-rTsMFas2 antibody (1:2000 dilution). Immunoreactive signals were developed using ECL after 2 min exposure. *Abbreviations*: *M*_r_, molecular weight in kDa; p*I*, isoelectric point

We determined the expression patterns of Fas proteins in different anatomical compartments of TsM and adult worms. Both proteins were ubiquitously detected during the metacestode and adult stages, with abundant expression patterns in the scolex and neck (Fig. 2a). The proteins were also observable in other compartments, including the bladder wall of the TsM and immature/mature/gravid proglottids of the adult worm. Interestingly, secretory behaviour of Fas1 and 2 proteins substantially differed among developmental stages. Large amounts of proteins were secreted during the metacestode stage into either CF or ESP, but relatively small amounts of proteins were secreted outside the parasite as the worm grew into an adult (Fig. 2a).

When we analyzed protein profiles of Fas1 and 2 extracted from SN by 2-DE immunoblotting, as shown in Fig. 2b, at least six isoforms of each protein with different p*I* values were detected (*M*_r_ of 85 kDa with p*I*s of 5.5–6.3 for Fas1 and *M*_r._ of 63 kDa with p*I*s of 5.4–6.4 for Fas2). Those isoforms might be produced by different types and/or degrees of phosphorylation (Additional file 1: Figure S1).

**Fig. 3:**
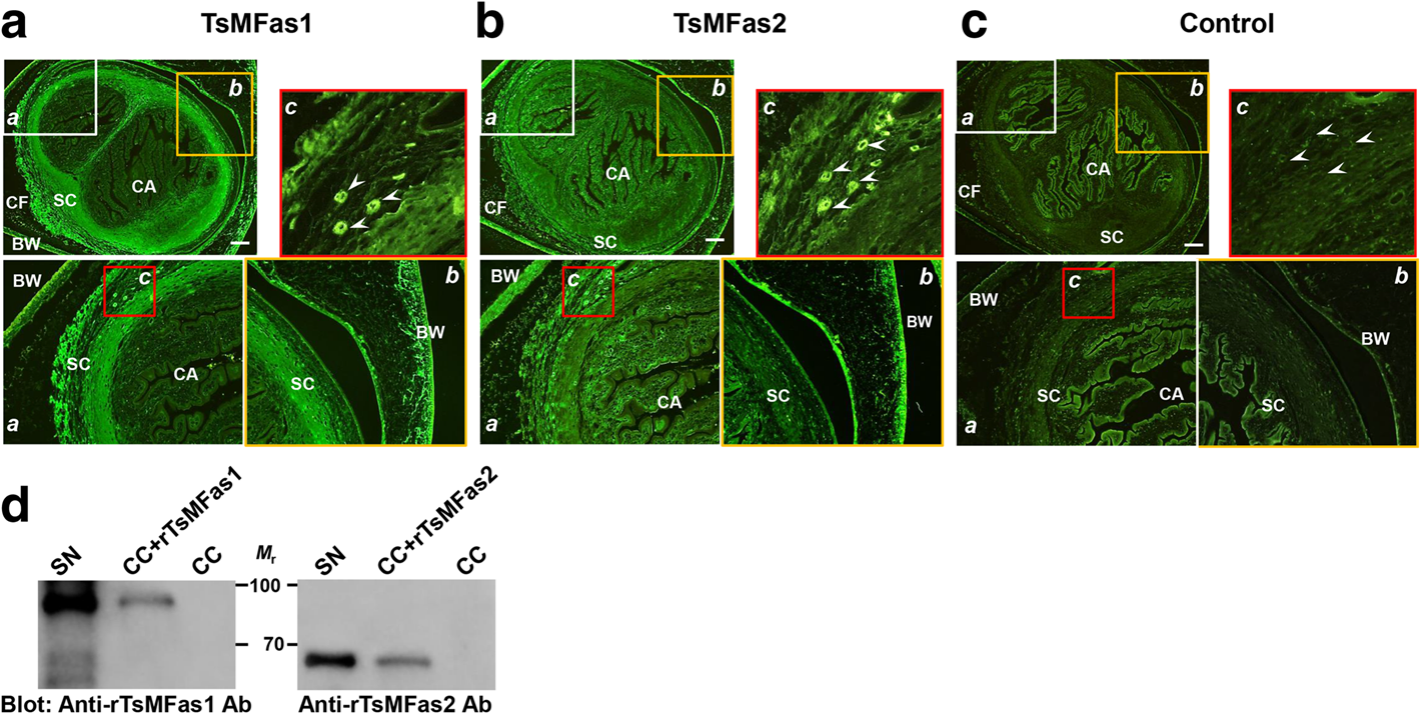
Immunohistochemical localization of Fas1 and Fas2 proteins in *T. solium* metacestode (TsM). **a**, **b** TsM sections (4 μm thick) were incubated with anti-rTsMFas1 or anti-rTsMFas2 antibody (1:200 dilution) and further incubated with FITC-conjugated anti-mouse IgG antibody (1:500 dilution). Areas marked by *a*, *b* and *c* are also shown in highlight views. White arrowheads show positive reactions to calcareous corpuscles (CC). *Abbreviations*: BW, bladder wall; CA, spiral canal; CF, cyst fluid; SC, scolex. *Scale-bars*: 200 μm. **c** TsM sections were probed with control IgG isolated from preimmune mouse serum (1:200 dilution) and subsequently with FITC-conjugated anti-mouse IgG antibody (1:500 dilution). Markings are the same as described in *a*. **d** In vitro binding of rTsMFas1 or rTsMFas2 with CC. rTsMFas 1 or 2 protein (each 10 μg) was incubated with CC (10 μl). The binding complex was precipitated, resuspended in 2× reducing sample buffer and separated by 8% SDS-PAGE. Proteins were transblotted to nitrocellulose membranes and probed with respective antibodies (1:2000 dilution). Immune signals were detected by ECL after 2 min exposure. Lane SN: scolex/neck proteins (10 μg); Lane CC + rTsMFas1 or 2: purified CC (each 10 μl) was incubated with rTsMFas1 or 2 (10 μg each); Lane CC: calcareous corpuscles only (10 μl). *Abbreviation*: *M*_r_, molecular weight in kDa

We subsequently determined tissue locality of Fas1 and 2 proteins. Specific antibodies against rTsMFas1 or 2 exhibited positive reactions to the parenchymal portion of the scolex with a diffuse fashion. Bladder wall also showed reaction pattern similar to that of scolex (panels *a* and *b*, Fig. 3a, b), which corroborated with immunoblotting findings (Fig. 2a). Notably, CCs scattered within the cellular parenchyma demonstrated strong positive reactions with anti-rTsMFas1 or 2 antibody (panel *c*, Fig. 3a, b). Worm sections treated with preimmune mouse serum did not exhibit any detectable positive reaction (Fig. 3c). We confirmed whether CCs interacted with Fas proteins by incubating the purified CC with rFas1 or 2 proteins. The bound protein was separated by SDS-PAGE, transblotted to nitrocellulose membrane and probed with anti-rTsMFas1 or 2 antibody. Each antibody was strongly reactive with corresponding Fas proteins (Fig. 3d). These results indicated that TsMFas1 and 2 specifically bound to CC and that TsMFas1 and 2 proteins did not share immunological cross-reactivity.

### Identification of TsM proteins bound to CC

**Fig. 4:**
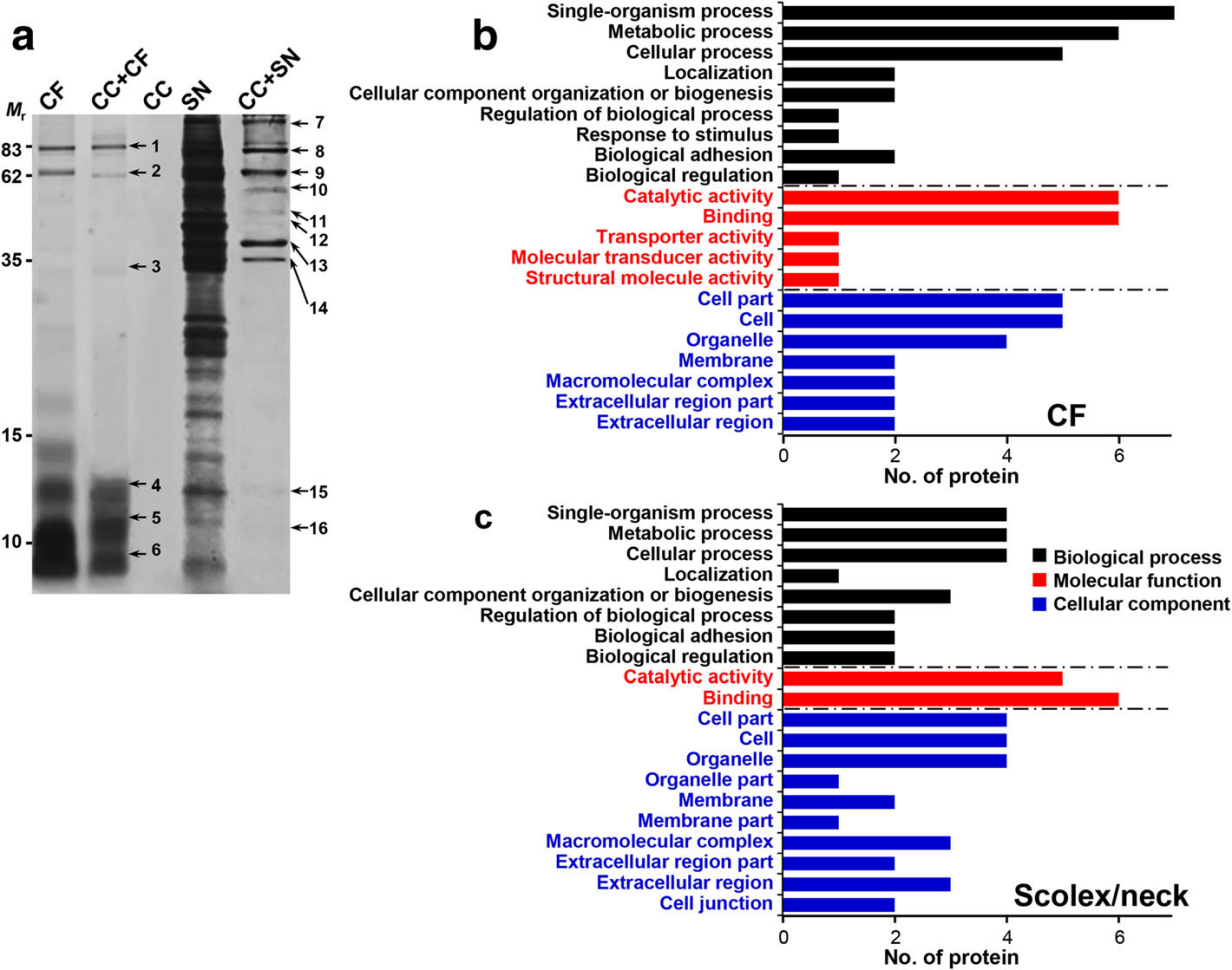
Identification of calcareous corpuscle (CC) binding proteins of *T. solium* metacestode (TsM). **a** SDS-PAGE analysis of protein repertoire of CC. CC was purified from TsM cellular compartments using a Ficoll-Plaque gradient sedimentation. Cyst fluid (CF) and scolex/neck (SN) proteins (10 μg each) were incubated with the purified CC (10 μl). The CC-protein complex was washed with PBS, precipitated by centrifugation and resuspended in 2× SDS-PAGE sample buffer. Proteins were separated by 15% SDS-PAGE under reducing conditions. The gel was stained with CBB. Binding partners (marked by 1–16) were subjected to protein identification by LC-ESI-MS/MS. Identified proteins are listed in Additional file 3: Table S1. Lane CF: cyst fluid (10 μg) only; Lane CC + CF: CC (10 μl) was incubated with CF (10 μg); Lane CC: calcareous corpuscle only (10 μl); Lane SN: scolex/neck protein only (10 μg); Lane CC + SN: CC (10 μl) was incubated with SN protein (10 μg). *Abbreviation: M*_r._ molecular weight in kDa. Functional categorization of identified proteins from CF (**b**) or scolex/neck (**c**). Gene ontology terms assigned to the biological process, molecular function and cellular component were analyzed by Blast2GO on the basis of similarity pattern employing the second-level of GO hierarchy [28]. The number of identified proteins in each functional group is shown in histogram

Previous studies have demonstrated that TsM CC bound to several molecules and suggested that CC might be involved in trafficking of those proteins [20–23]. We analyzed the protein ligands of CC with CF or cytosolic proteins extracted from SN. As shown in Fig. 4a, at least six CF and 10 SN protein bands appeared to bind to CC by SDS-PAGE analysis. A total of 41 proteins were identified by LC-ESI-MS/MS analysis, among which 24 proteins represented distinctive species (Additional file 3: Table S1). Out of 14 protein ligands detected in cellular proteins (SN extracts), five species (paramyosin, actin, innexin unc-9, enolase and phosphoglycerate kinase 1 [PGK1]) were uniquely recognized. Ten CC-binding CF proteins, such as four different enzymes (glycogen phosphorylase, lysyl oxidase, malate dehydrogenase and aldo keto reductase), two ECM proteins (basement membrane specific heparan sulphate [HPGS] and collagen α1XV chain) and transporter (lipid transport protein and major vault protein), were identified. One each of cellular protection protein (heat shock protein 83) and expressed protein (TsM_000826300) was also detected in CF. Eight distinct protein ligands were discovered in both CF and SN proteins. These proteins were two secreted glycoprotein antigens (antigen B-like proteins and secreted antigen Ts8B1), two species of carbohydrate metabolizing enzymes (glyceraldehyde 3-phosphate dehydrogenase [GAPDH] and phosphoenolpyruvate carboxykinase [PEPCK]), N-acetylated α-linked acidic dipeptidase 2 (NAALAD2) and an expressed protein (TsM_000414400) (Additional file 3: Table S1). Fas1 and 2 were also found to be a repertoire of CC in both CF and SN proteins (band nos. 1, 2, 8 and 9, Fig. 4a), which matched well with results of immunohistochemical staining and in vitro binding assay (Fig. 3).

A systematic analysis employing gene ontology terms were used to assign identified proteins based on similarity patterns. Both CF and SN proteins that bound to CC were largely categorized into the biological process (single-organism process, metabolic process and cellular process) and cellular component (cell part, cell and organelle). Molecular functions were restricted to binding and catalytic activity (Fig. 4b, c). These results indicated that the protein ligands of CC might be mostly associated with the cellular process including cell-cell interactions and metabolic processes.

### Protein-protein interactions associated with CC-Fas1 or CC-Fas2 binary complex

Protein ligands from the cellular fraction that bound to CC and Fas1 or Fas2 protein complex were further analyzed. Prior to the determination of protein repertoires, we confirmed whether CC-Fas binary complex indeed played a major role during protein-protein interactions because TsM CC was able to bind several molecules including Fas1 and 2 proteins (lane CC + SN, Fig. 4a). Endogenous Fas proteins existed in cellular fractions might have effects on binding characteristics.

**Fig. 5:**
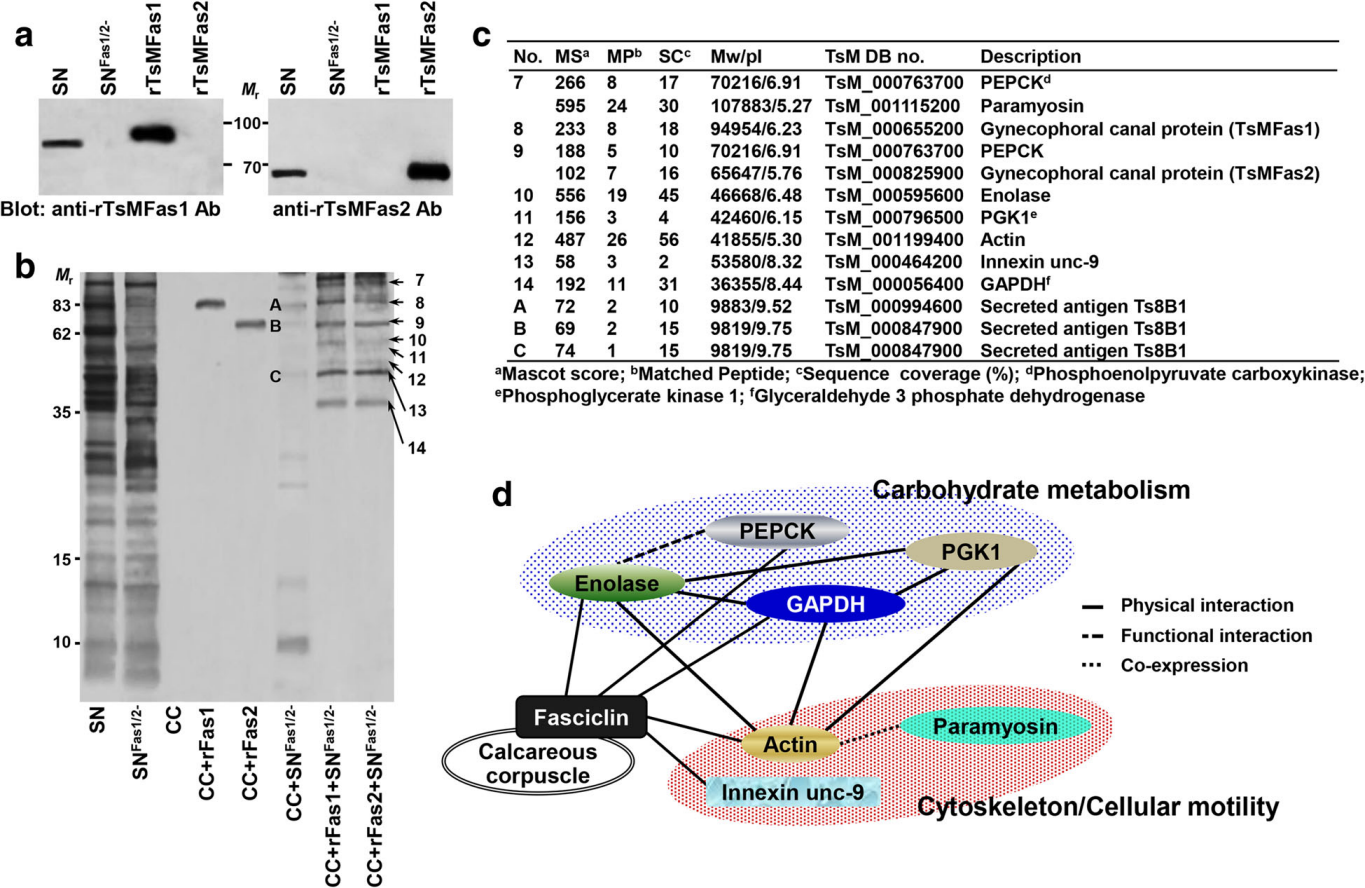
Identification of *T. solium* metacestode (TsM) cellular proteins bound to calcareous corpuscle (CC)-fasciclin (TsMFas1 or TsMFas2) binary complex. **a** Immunoblot analysis of scolex/neck (SN) proteins depleted of TsMFas1/2 proteins. SN proteins were incubated with protein G-coupled anti-rTsMFas1/2 antibodies, and unbound proteins were eluted in flow-through fractions. Proteins (10 μg) were separated by 8% SDS-PAGE under reducing conditions and transblotted to a nitrocellulose membrane. Blots were probed with anti-rTsMFas1 or anti-rTsMFas2 antibody (1:2000 dilution). Immunoreactive signals were developed using ECL after 2 min exposure. Lane SN: whole SN proteins; Lane SN^Fas1/2-^: SN protein depleted of Fas1/2. *Abbreviation: M*_r._, molecular weight in kDa. **b** TsM SN proteins depleted of Fas1 and Fas2 proteins (10 μg) were incubated with CC-rTsMFas1 or CC-rTsMFas2 binary complex (each 10 μl) and precipitated by centrifugation. Pellets were resuspended in 2× SDS-PAGE reducing sample buffer, separated on 15% gels and stained with CBB. Lane SN: scolex/neck protein (10 μg); Lane SN^Fas1/2-^: SN protein depleted of Fas1/2 (10 μg); Lane CC: purified calcareous corpuscle (10 μl) only; Lane CC + rFas1 or 2: purified CC was incubated with rFas1 or rFas2 protein; Lane CC + SN^Fas1/2-^: Fas1/2 depleted SN proteins were incubated with CC; Lane CC + rFas1/2 + SN^Fas1/2-^: CC-rFas1 or CC-rFas2 binary complex was incubated with Fas1 and Fas2 depleted SN proteins. *Abbreviation: M*_r._, molecular weight in kDa. **c** Identification of protein repertoire for CC-Fas complex. Each protein band (7–14 and A-C) was processed for protein identification by LC-ESI-MS/MS. Independent duplicated biological samples were analysed. **d** Construction of protein-protein interaction network mediated by CC-Fas1 or CC-Fas2 complex by STRING algorithm ver10.0 (http://string-db.org/). Correlated interactions extracted from the platyhelminth proteins are presented with their predicted functional partners

We depleted Fas1 and 2 molecules from cellular proteins through protein G-immunoaffinity chromatography coupled with anti-rTsMFas1 and 2 antibodies (Fig. 5a). When binding properties of this fraction with CC were assessed, significantly reduced binding partners were observed compared to those with CC-Fas binary complex (compare binding partners between lanes CC + SN^Fas1/2-^ and CC + rFas1 + SN^Fas1/2-^, Fig. 5b). Moreover, Fas1/2 depleted SN extracts bound to CC revealed different protein repertoires based on LC-ESI-MS/MS analysis. The three bands (marked by A-C), which showed similar *M*_r._ with those bound to CC-Fas binary complex, were identified to be a secretory antigen Ts8B1 (TsM_000847900) (Fig. 5c). This result demonstrated that Fas proteins might play critical roles in protein-protein interaction.

As shown in Fig. 5b (lanes CC + rFas1 + SN^Fas1/2-^ and CC + rFas2 + SN^Fas1/2-^), at least eight protein bands were interactive with the CC-TsMFas binary complex (band nos. 7–14). CC-TsMFas1 or CC-TsMFas2 complex had the same binding partners. Those binding proteins comprised of 10 distinct species. Carbohydrate metabolizing enzymes such as enolase, PEPCK, PGK1 and GAPDH were found to be protein ligands. In addition, proteins associated with cytoskeleton and cellular motility, including paramyosin, actin and innexin unc-9, were identified (Fig. 5c). In silico mapping for integrated protein-protein interaction using STRING ver10 algorithm demonstrated that those proteins had direct (physical) and indirect (functional) associations in accordance with their biological and/or biochemical properties (Fig. 5d).

## Discussion

This study characterized molecular properties and biological roles of TsMFas proteins. Multiple isoforms of two paralogous TsMFas1 and TsMFas2 proteins which might be produced by post-translational modifications were expressed abundantly in the cellular compartments of the worm during metacestode and adult stages. The proteins showed adhesive properties with other cells. Immunoblot analysis and tissue localization demonstrated that TsMFas molecules might interact with CC and other cellular proteins. We determined, for the first time, integrated protein-protein interactions mediated by CC-Fas binary complex.

Calcareous corpuscles of the platyhelminths are not stationary concretions since they form, organize/reorganize and become resorbed in a given host [31]. Cestode parasites possess significant amounts of CC, comprising up to 40% of the dry weight of the organism [32]. The presence of huge quantity and maintenance of the physical integrity through continuous remodelling suggest that the organelle might be associated with cellular processes inherent to parasite physiology. To better understand symbiotic protein interactions possibly mediated by CC, we analyzed the secretory protein (CF) and parenchymal cytosolic proteins (scolex/neck extracts) bound to CC. We were able to identify 19 secretory and 14 cellular proteins bound to CC.

When we analyzed the protein identities of those repertoires, multiple protein ligands involved in diverse cellular processes and metabolic pathways were detected. The major proportions were segregated into carbohydrate metabolism related proteins (enolase, GAPDH, malate dehydrogenase, PGK1 and PEPCK) and cytoskeletal/cell motility proteins (paramyosin, actin and innexin unc-9). In addition, low-molecular weight proteins, which induce specific antibody responses with patient sera of active stage NC [5, 33, 34], were recognized. Most of these ligands shared common properties of calcium dependency [35–37] and/or post-translationally modified by glycosylation [8]. Previous studies have reported that cestode CC bound to calcium binding proteins [20, 23] and glycoproteins [21, 23]. Calcium-related biochemical properties of TsMFas1 and TsMFas2 proteins were not examined in this study. However, we thought that biomineral calcium deposited in CC might be involved in binding of TsMFas1/2 molecules with CC.

Fas1-domain-containing proteins participate in important biological events during parasite development by regulating cellular processes including adhesion, migration and differentiation of muscle cells and sexual maturation of reproductive system [11, 14, 15]. The proteins also play a role in targeting of axon-generating neuronal cells, which might lead to the development of nervous system complexity in insects [38]. In this study, two paralogous TsMFas proteins showed significant molecular divergence (Fig. 1b and Additional file 1: Figure S1), which implicated that these proteins might have different sub-functions. Interestingly, however, these two TsMFas proteins demonstrated similar expression patterns and co-localized with CC (Fig. 3a, b). The binding assay also revealed that rTsMFas1/2 could bind to CC (Fig. 3d). These results collectively indicate that TsMFas1/2 proteins might constitute a set of ligands for CC. Those observations prompted the current analysis of CC-Fas binary complex mediated protein-protein interactions, which resulted in the detection of several protein ligands (Fig. 5b). When we analyzed domain organization of those protein ligands, only TsMFas1/2 proteins possessed Fas-related domains. Another fasciclin 1 protein harbouring Fas1-domain was found in the *T. solium* GeneDB (TsM_000180200), but this protein was not a binding partner of CC (Fig. 4a, 5b and Additional file 3: Table S1). This result strongly suggests that the ligands might bind to the complex via Fas1-domain independent manner. This result also implies that biological roles engaged in TsM_000180200 protein might be different from TsMFas1/2 proteins characterized in this study. Which motifs/domains of these protein ligands are involved in the binding are currently unknown. This intriguing issue deserves further study.

TsMFas proteins were shown to be secreted through classical pathway because they possessed a signal peptide. However, large proportions of TsMFas proteins were localized in cellular parenchymal regions (Figs. 2, 3) and suggest that biological relevance of TsMFas proteins might be deeply related to the cellular parenchyma. Significantly decreased secretion of Fas molecules in adult compared to that in metacestode stage (Fig. 2a) also suggested functional roles of Fas proteins might be specifically elaborated in the regulation of cellular biological processes. When a metacestode matures into an adult, the parasite’s body length is enlarged more than 100 times, and the body compartments are transformed mostly into the cellular parenchyma. This metamorphic change might require more Fas proteins to supply sufficient amounts of proteins to meet and maintain cellular demands, which might subsequently decrease the secretory behaviour of Fas molecules. Moreover, *T. solium* adult worm does not bestow to host tissue but thrives in the intestinal lumen. Conversely, large quantities of Fas1/2 molecules secreted into surrounding environments during the metacestode stage might exert effects when worms are attached to host tissues.

When we analyzed protein-protein interactions mediated by CC-Fas binary complex, similar protein repertoires were observed compared to those of CC only. We surmised that this observation might be attributable to TsM parenchymal extracts used in binding assay. The TsM native cellular proteins contained significant amounts of endogenous Fas1 and Fas2 (Fig. 2). These Fas molecules would bind to CC and influenced the binding characteristics. We depleted Fas1 and Fas2 molecules from cellular proteins through immunoaffinity chromatography, and such proteins were used in binding assay. The numbers of binding molecules and binding intensities were remarkably reduced, and different proteins were found to bind to CC only (Fig. 5b, c). This result demonstrated that Fas1 and Fas2 molecules were indeed intimately involved in protein-protein interactions. In addition, protein ligands were more restricted to molecules involved in metabolic pathways and ECM components. Most of the low molecular weight antigenic proteins did not bind to the CC-Fas binary complex. This result also supported the notion that protein networks mediated by CC-Fas binary complex seemed to be more specific for the cellular physiology of the worm.

CC-Fas complex binding partners were categorized into two major groups: carbohydrate metabolizing enzymes and proteins constituting cytoskeleton and cell motility (Fig. 5d). When we observed relationships of those protein ligands by in silico STRING analysis, enzymes involved in carbohydrate metabolism such as enolase, PEPCK, GAPDH and PGK1 showed direct (physical) and/or indirect (functional) relationships. Glycolysis/gluconeogenesis is a key metabolism essential for parasite survival. Glucose constitutes the main resource for supplying energy because biogenetic pathways exploited by helminths occur mainly under anaerobic conditions, where tricarboxylic acid (TCA) cycle and respiratory chains are markedly limited [39]. Imbalanced glucose metabolism causes several cellular defects in response to anoxic conditions and induces germ cell apoptosis in the free living nematode, *Caenorhabditis elegans* [40, 41]. The establishment and maintenance of a symbiotic system for carbohydrate metabolism might be important for an efficacious parasitic way of life.

The second group of ligands was cytoskeletal and cell motility proteins such as actin, paramyosin and innexin unc-9. Actin and paramyosin are dynamic ECM components involved in numerous cell biological processes in eukaryotes, which include cytogenesis, cytoplasmic organization, cellular motility and endocytosis [42]. TsM paramyosin, also known as antigen B (AgB), is distributed in the tegument with collagen binding-activity [43]. This protein interaction was functionally associated with CC-Fas-actin network. Innexin unc-9 is a gap junction protein uniquely found in low invertebrates. The protein is widely expressed in motor neurons and muscular intracellular junctions of *C. elegans*. It plays a role in cellular mobility through regulating low conductance gap junctions and synaptic plasticity [44, 45]. Our results demonstrated that the protein concomitantly binds to CC-Fas complex. This functional network might constitute an effective locomotive system for parasitic motility.

## Conclusions

In this study, we characterized two paralogous *T. solium* fasciclin proteins. Multiple isoforms of the proteins are abundantly expressed in the cellular parenchyma of metacestode and adult stages. The proteins are co-localized with CC scattered in the parenchyma. An in vitro binding assay demonstrated that the CC-Fas binary complex might function in matrix-mediated symbiotic protein interactions. The binding partners mostly represent enzymes involved in carbohydrate metabolism and proteins composing cytoskeleton/cellular motility. The protein networks may contribute to the long-term survival of the parasite by maintaining worm’s homeostatic functions and might be crucial for the pathobiology of NC. Further studies are warranted to elucidate their cooperative and/or synergistic activities. NC is endemic in several countries and causes significant public health burdens. To combat against NC, targeting of protein networks associated with important metabolic pathway and motility might be useful for the development of novel chemotherapeutics or vaccines.

## Additional files

Additional file 1: Figure S1. Comparison of primary structures of TsMFas1 and TsMFas2. Dashes represent gaps introduced into sequences to maximize sequence identity during alignment. Recognition sites for signal peptidase are shown by arrows. Theoretical serine/threonine and tyrosine phosphorylation sites are indicated by red letters. N-glycosylation sites are denoted by blue letters while O-glycosylation sites are marked with open circles. Fasciclin-domain (green boxes) and fasciclin-superfamily domain (green-dotted boxes) are indicated. Highly-conserved domains of H1 (red lines) and H2 (dotted red line) found in Fas1-like molecules are also shown. Phosphorylation, N-glycosylation and O-glycosylation sites were predicted by NetPhos 3.1 (http://www.cbs.dtu.dk/services/NetPhos/), NetNGlyc 1.0 (http://www.cbs.dtu.dk/services/NetNGlyc/) and NetOGlyc 4.0 (http://www.cbs.dtu.dk/services/NetOGlyc/), respectively. (TIFF 711 kb)

Additional file 2: Figure S2. Efficiency of rTsMFas1 and rTsMFas2 in cellular adhesion. **a** Expression and purification of recombinant proteins. Bacterially expressed rTsMFas1 and rTsMFas2 proteins were purified using Ni-NTA column, after which His-tag was removed by thrombin cleavage. Proteins (each 200 ng) were monitored with 8% reducing SDS-PAGE followed by CBB staining. **b** Each well of a 96-well plate was coated with BSA (2 μg/ml), fibronectin (10 μg/ml) and each recombinant protein (10 μg/ml), after which incubated with MRC-5 and NHLF cells. Attached cells were measured by the hexosamidase assay. Graphic values of average and error bars representing standard deviations were obtained from triplicate assays of three independent experiments. (TIFF 220 kb)

Additional file 3 **Table S1.** Identification of calcareous corpuscle binding TsM proteins by LC-ESI-MS/MS. (DOCX 22 kb)

## Abbreviations

2-DE: 2-dimensional gel electrophoresis
CC: Calcareous corpuscle
CF: Cyst fluid
DALY: Disability-adjusted life years
DTT: Dithiothreitol
ECL: Enhanced chemiluminescence
ECM: Extracellular matrix
ESP: Excretory-secretory products
Fas: Fasciclin
FITC: Fluorescein isothiocyanate
GAPDH: Glyceraldehyde 3-phosphate dehydrogenase
GCP: Gynecophoral canal protein
HRP: Horse-radish peroxidase
MRC-5: Human fetal lung fibroblast
NAALAD2: N-acetylated α-linked acidic dipeptidase 2
Nano-LC-ESI-MS/MS: Nano-liquid chromatography-electrospray ionization-multistage mass spectrometry
NC: Neurocysticercosis
NHLF: Normal human lung fibroblast
PBS: Phosphate buffered saline
PBS/T: PBS containing 0.05% Tween 20
PEPCK: Phosphoenolpyruvate carboxykinase
PGK1: Phosphoglycerate kinase 1
rTsMFas1/2: Recombinant TsM Fas1/2
SN: Scolex and neck
TsM: *Taenia solium* metacestode
TsMFas: TsM fasciclin
