# Supplementary figures and images for: Fasciclin-calcareous corpuscle binary complex mediated protein-protein interactions in Taenia solium metacestode
Source: Parasit Vectors. 2017 Sep 20;10:438. doi: 10.1186/s13071-017-2359-2 (PMC5606126; doi:10.1186/s13071-017-2359-2)

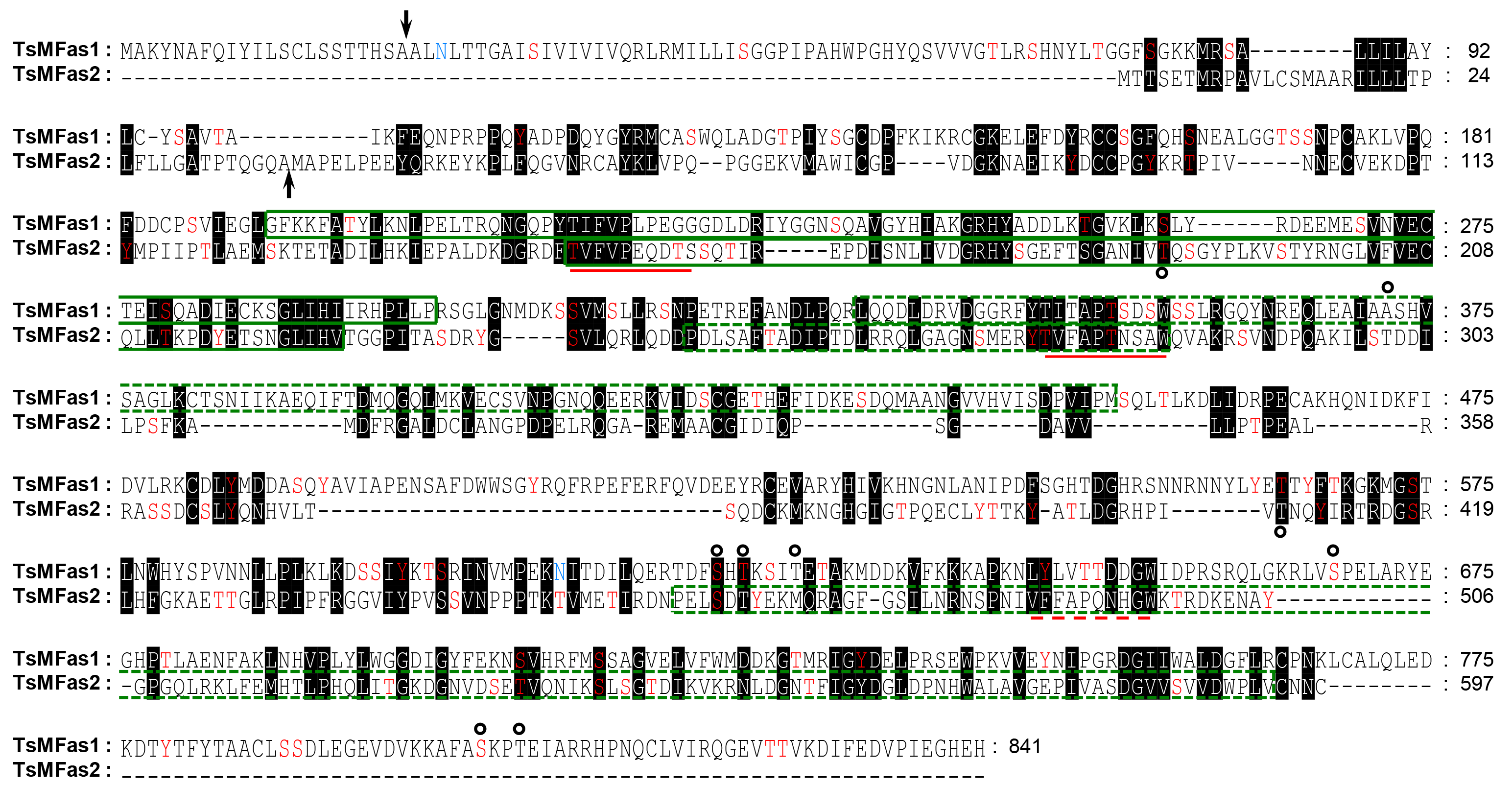

Supplement: Supplementary file 1 — Comparison of primary structures of TsMFas1 and TsMFas2. Dashes represent gaps introduced into sequences to maximize sequence identity during alignment. Recognition sites for signal peptidase are shown by arrows. Theoretical serine/threonine and tyrosine phosphorylation sites are indicated by red letters. N-glycosylation sites are denoted by blue letters while O-glycosylation sites are marked with open circles. Fasciclin-domain (green boxes) and fasciclin-superfamily domain (green-dotted boxes) are indicated. Highly-conserved domains of H1 (red lines) and H2 (dotted red line) found in Fas1-like molecules are also shown. Phosphorylation, N-glycosylation and O-glycosylation sites were predicted by NetPhos 3.1 (http://www.cbs.dtu.dk/services/NetPhos/), NetNGlyc 1.0 (http://www.cbs.dtu.dk/services/NetNGlyc/) and NetOGlyc 4.0 (http://www.cbs.dtu.dk/services/NetOGlyc/), respectively. (TIFF 711 kb) [file 13071_2017_2359_MOESM1_ESM.tif]

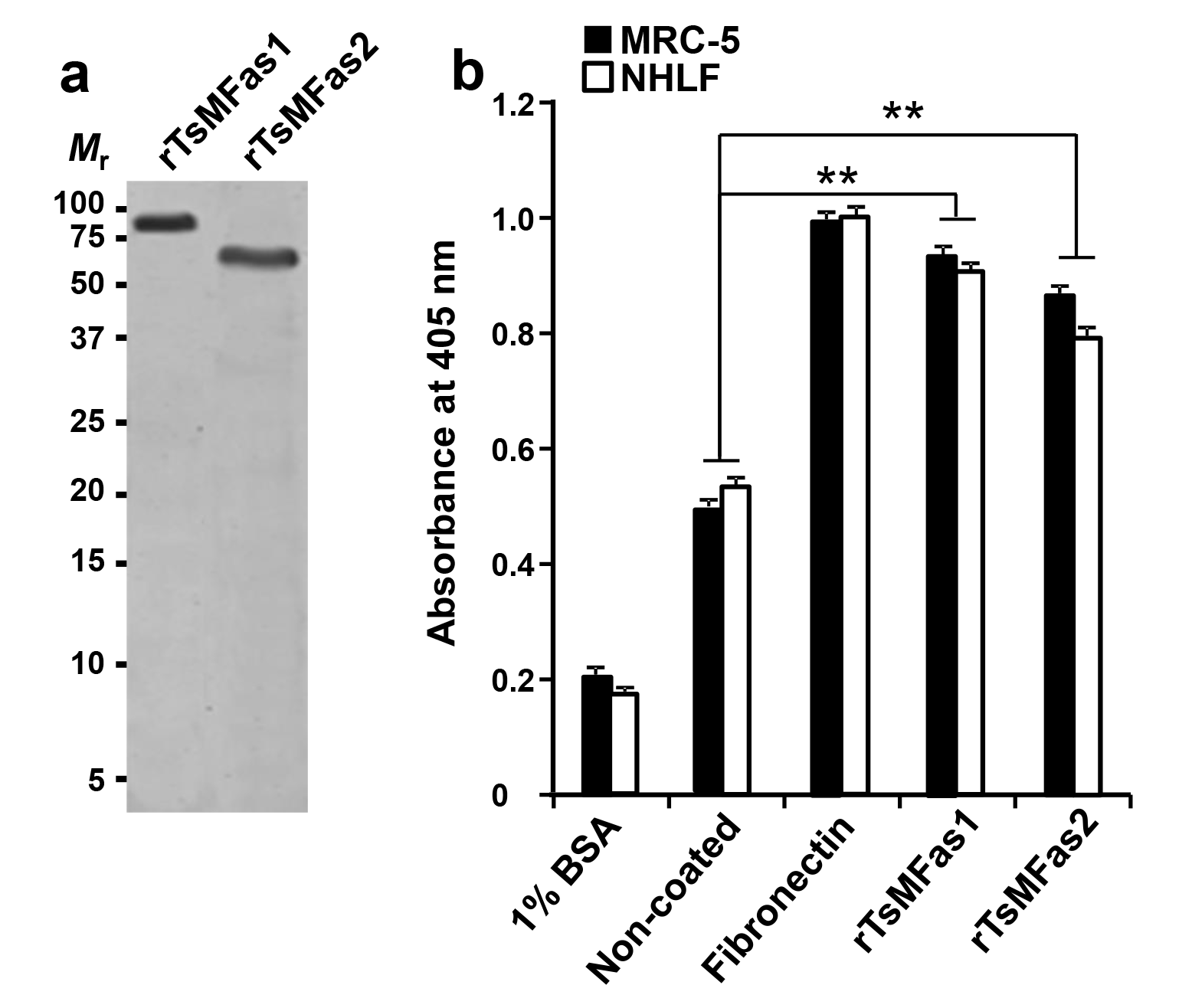

Supplement: Supplementary file 2 — Efficiency of rTsMFas1 and rTsMFas2 in cellular adhesion. a Expression and purification of recombinant proteins. Bacterially expressed rTsMFas1 and rTsMFas2 proteins were purified using Ni-NTA column, after which His-tag was removed by thrombin cleavage. Proteins (each 200 ng) were monitored with 8% reducing SDS-PAGE followed by CBB staining. b Each well of a 96-well plate was coated with BSA (2 μg/ml), fibronectin (10 μg/ml) and each recombinant protein (10 μg/ml), after which incubated with MRC-5 and NHLF cells. Attached cells were measured by the hexosamidase assay. Graphic values of average and error bars representing standard deviations were obtained from triplicate assays of three independent experiments. (TIFF 220 kb) [file 13071_2017_2359_MOESM2_ESM.tif]
