# Supplementary material for: Fasciclin-calcareous corpuscle binary complex mediated protein-protein interactions in Taenia solium metacestode
Source: Parasit Vectors. 2017 Sep 20;10:438. doi: 10.1186/s13071-017-2359-2 (PMC5606126; doi:10.1186/s13071-017-2359-2)
Supplement: Supplementary file 3 — Table S1. Identification of calcareous corpuscle binding TsM proteins by LC-ESI-MS/MS. (DOCX 22 kb) [file 13071_2017_2359_MOESM3_ESM.docx]

**Additional file 3:** Table S1. Identification of calcareous corpuscle binding TsM proteins by LC-ESI MS/MS.

| Band no. | MS^a^ | MP^b^ | SC^c^ | Theoretical  Mw/pI | TsM DB no. (TsM_) | Predicted protein | CD^d^ |
| --- | --- | --- | --- | --- | --- | --- | --- |
| 1 | 2290 | 94 | 47 | 87599/8.70 | 000691600 | NAALAD2 | CF/CP |
|  | 2081 | 88 | 22 | 341824/6.93 | 000130500 | Lipid transport protein | CF |
|  | 448 | 23 | 20 | 97080/5.47 | 000555100 | Major vault protein | CF |
|  | 445 | 15 | 14 | 93421/5.06 | 001048700 | HSPG | CF |
|  | 240 | 15 | 37 | 9732/9.19 | 000374400 | Antigen B like protein | CF/CP |
|  | 116 | 5 | 8 | 73205/8.71 | 000970200 | Glycogen phosphorylase | CF |
|  | 98 | 4 | 22 | 9819/9.75 | 000847900 | Secreted antigen Ts8B1 | CF/CP |
|  | 80 | 4 | 6 | 78506/5.08 | 000762300 | Heat shock protein 83 | CF |
|  | 85 | 3 | 10 | 9883/9.52 | 000994600 | Secreted antigen Ts8B1 | CF/CP |
|  | 84 | 3 | 4 | 94954/6.23 | 000655200 | GCP (TsMFas1) | CF/CP |
| 2 | 1145 | 47 | 48 | 70171/6.91 | 000763700 | PEPCK | CF/CP |
|  | 290 | 15 | 37 | 36355/8.44 | 000056400 | GAPDH | CF/CP |
|  | 96 | 3 | 6 | 56579/5.91 | 000635400 | Lysyl oxidase | CF |
|  | 88 | 3 | 6 | 65647/5.76 | 000825900 | GCP (TsMFas2) | CF/CP |
|  | 80 | 4 | 5 | 67327/8.64 | 000826300 | Expressed protein | CF |
| 3 | 256 | 12 | 32 | 36628/8.10 | 000048200 | Malate dehydrogenase | CF |
|  | 151 | 8 | 34 | 9732/9.19 | 000374400 | Antigen B like protein | CF/CP |
|  | 111 | 5 | 18 | 20682/7.66 | 000066800 | Collagen α1 (XV) chain | CF |
|  | 73 | 2 | 3 | 35561/6.80 | 000353300 | Aldo keto reductase | CF |
| 4 | 165 | 12 | 44 | 9732/9.19 | 000374400 | Antigen B like protein | CF/CP |
|  | 127 | 11 | 27 | 9883/9.52 | 000994600 | Secreted antigen Ts8B1 | CF/CP |
|  | 106 | 1 | 14 | 14117/5.74 | 000414400 | Expressed protein | CF/CP |
|  | 88 | 2 | 15 | 9819/9.75 | 000847900 | Secreted antigen Ts8B1 | CF/CP |
| 5 | 104 | 3 | 23 | 9819/9.75 | 000847900 | Secreted antigen Ts8B1 | CF/CP |
|  | 83 | 2 | 9 | 9732/9.19 | 000374400 | Antigen B like protein | CF/CP |
| 6 | 61 | 2 | 18 | 9883/9.52 | 000994600 | Secreted antigen Ts8B1 | CF/CP |
| 7 | 595 | 24 | 30 | 107883/5.27 | 001115200 | Paramyosin | CP |
|  | 266 | 8 | 17 | 70216/6.91 | 000763700 | PEPCK | CF/CP |
| 8 | 233 | 8 | 18 | 94954/6.23 | 000655200 | GCP (TsMFas1) | CF/CP |
|  | 128 | 4 | 9 | 87765/8.70 | 000691600 | NAALAD2 | CF/CP |
| 9 | 188 | 5 | 10 | 70216/6.91 | 000763700 | PEPCK | CF/CP |
|  | 102 | 7 | 16 | 65647/5.76 | 000825900 | GCP (TsMFas2) | CF/CP |
| 10 | 556 | 19 | 45 | 46668/6.48 | 000595600 | Enolase | CP |
| 11 | 156 | 3 | 4 | 42460/6.15 | 000796500 | Phosphoglycerate kinase 1 | CP |
| 12 | 487 | 26 | 56 | 41855/5.30 | 001199400 | Actin | CP |
| 13 | 58 | 3 | 2 | 52580/8.32 | 000464200 | Innexin unc-9 | CP |
| 14 | 192 | 11 | 31 | 36355/8.44 | 000056400 | GAPDH | CF/CP |
| 15 | 106 | 2 | 12 | 14117/5.74 | 000414400 | Expressed protein | CF/CP |
|  | 71 | 3 | 22 | 9883/9.52 | 000994600 | Secreted antigen Ts8B1 | CF/CP |
| 16 | 285 | 18 | 40 | 9732/9.19 | 000374400 | Antigen B like protein | CF/CP |
|  | 102 | 3 | 25 | 9819/9.75 | 000847900 | Secreted antigen Ts8B1 | CF/CP |

^a^Mascot score. Protein scores are derived from ions scores. Ions score is -10×Log(P), where P is the probability that the observed match is a random event. Individual ions scores > 37 indicate identity or extensive homology (*P* < 0.05).

^b^Matched peptide

^c^Sequence coverage (%)

^d^Cellular distribution. Cellular distributions were divided into cyst fluid (CF) and cellular parenchyma (CP).

*Abbreviations:* GAPDH: glyceraldehyde 3-phosphate dehydrogenase; GCP: gynecophoral canal protein; HSPG: basement membrane specific heparan sulfate; NAALAD2: N-acetylated alpha-linked acidic dipeptidase 2; PEPCK: phosphoenolpyruvate carboxykinase.
